# A randomized clinical study on the impact of Comprehensive Geriatric Assessment (CGA) based interventions on the quality of life of elderly, frail, onco-hematologic patients candidate to anticancer therapy: protocol of the ONCO-Aging study

**DOI:** 10.1186/s12877-021-02237-3

**Published:** 2021-05-19

**Authors:** Abdurraouf Mokhtar Mahmoud, Federica Biello, Paola Maria Maggiora, Riccardo Bruna, Giovanni Burrafato, Miriam Cappelli, Feba Varughese, Veronica Martini, Francesca Platini, Clara Deambrogi, Andrea Patriarca, Maura Nicolosi, Ajay ram Vachanaram, Carla Pisani, Eleonora Ferrara, Elvira Catania, Danila Azzolina, Francesco Barone-Adesi, Marco Krengli, Gianluca Gaidano, Alessandra Gennari

**Affiliations:** 1 Division of Oncology, Department of Translational Medicine, University of Eastern Piedmont, Azienda Ospedaliero-Universitaria Maggiore della Carità, Via Solaroli 17, 28100 Novara, Italy; 2 Division of Hematology, Department of Translational Medicine, University of Eastern Piedmont, Azienda Ospedaliero-Universitaria Maggiore della Carità, Novara, Italy; 3 Department of Palliative Care, Azienda Ospedaliero-Universitaria Maggiore della Carità, Novara, Italy; 4 Division of Radiation Oncology, Department of Translational Medicine, University of Eastern Piedmont, Azienda Ospedaliero-Universitaria Maggiore della Carità, Novara, Italy; 5 Unit of Medical Statistics, Department of Translational Medicine, University of Eastern Piedmont and CPO Piemonte, Novara, Italy; 6 Unit of Epidemiology, Department of Translational Medicine, University of Eastern Piedmont, Novara, Italy

**Keywords:** Comprehensive geriatric assessment (CGA), G8 questionnaire, Quality of Life (QoL), Cell senescence

## Abstract

**Background:**

Age is considered as one of the most important risk-factor for many types of solid and hematological cancers, as their incidence increases with age in parallel to the ever-growing elderly population. Moreover, cancer incidence is constantly increasing as a consequence of the increase in life expectancy that favors the process of cellular senescence. Geriatric assessment has been increasingly recognized as predictive and prognostic instrument to detect frailty in older adults with cancer. In particular, the G8 score is a simple and reproducible instrument to identify elderly patients who should undergo full geriatric evaluation. Due to their frailty, elderly patients may be often under-treated and a therapeutic choice based also on a comprehensive geriatric assessment (CGA) is recommended. With these premises, we aim to test the impact of the CGA based interventions on the quality of life (QoL) of frail elderly onco-hematological patients, identified by the G8 screening, candidate for innovative target directed drugs or treatments including the combination of radiotherapy and chemotherapy (RT + CT).

**Methods:**

Patients aged > 65 years, candidate to target directed agents or to RT + CT treatments are screened for frailty by the G8 test; those patients classified as frail (G8 ≤ 14) are randomized to receive a CGA at baseline or to conventional care. The primary endpoint is QoL, assessed by EORTC QLQ-C30C. As collateral biological study, the potential prognostic/predictive role of T-cell senescence and myeloid derived suppressor cells (MDSC) are evaluated on plasma samples.

**Discussion:**

This trial will contribute to define the impact of CGA on the management of frail elderly onco-hematologic patients candidate to innovative biological drugs or to integrated schedules with the association of RT + CT. Furthermore, the use of plasma samples to assess the potential prognostic value of imbalance of immune-competent cells is expected to contribute to the individualized care of elderly patients, resulting into a fine tuning of the therapeutic strategies.

**Trial registration:**

ClinicalTrials.gov ID: NCT04478916. registered July 21, 2020 – retrospectively registered.

## Background

Age is considered as one of the most important risk-factor for many types of solid and hematological cancers, as their incidence increases with age in parallel to the ever-growing elderly population [1]. The progressive increase of life expectancy facilitates the accumulation of cellular dysfunctions including “cell senescence”, indirectly favoring carcinogenesis [2, 3], because some of the mechanisms involved in aging are also involved in age-related diseases such as cancer [4].

Elderly patients often require tailored and different strategies compared to their younger counterparts [5–7]. In fact, aging is associated with decreased physiological reserves and altered pharmacokinetics, reducing treatment tolerability and enhancing the risk of treatment-related adverse events. Moreover, goals and priorities may indeed differ greatly according to age when making treatment decisions and preserving health-related quality of life (HRQoL).

It must be also taken into account that in the elderly onco-hematological patient, host dependent factors, such as those related to immunosenescence may interfere with treatment efficacy through the induction of immunosuppression mediated by myeloid derived suppressor cells (MDSC) in different inflammatory conditions [8–10]. Moreover, cellular senescence, a highly dynamic, multi-step process, during which the properties of senescent cells continuously evolve and diversify, may interfere with drug sensitivity, ending in an unfavorable prognosis [11, 12].

Recent guidelines by the American Society of Clinical Oncology (ASCO) strongly recommend a minimum core of geriatric assessment based on the evaluation of function, comorbidity, falls, depression, cognition, and nutrition in all patients who are candidates for oncological treatments [13]. Current guidelines by the Italian Association of Medical Oncology (AIOM) recommend the administration of a Comprehensive Geriatric Assessment (CGA) to all patients who are aged 70 years or older and who screen positive at screening tools such as the G8 or the Vulnerable Elders Survey-13 (VES-13) [14, 15].

In the perspective of a global care of the oncologic elderly patient, the use of a screening test for vulnerability represents a first step to streamline decisions: G8 has demonstrated such ability to identify patients requiring a dedicated CGA, with a threshold of ≤ 14/17 and a strong 1-year prognostic value [16].

Due to the possible complexity of their clinical conditions and comorbidities, elderly patients are often underrepresented in clinical trials; thus, a major challenge in oncology, as well as an unmet need, is to increase the relevance of clinical trials in older patients, to improve research in the field of geriatric oncology and to promote translational studies on the interface between aging and cancer.

We designed the ONCO-Aging study to fill this gap of knowledge; the primary objective is to optimize the care of elderly and frail onco-hematological patients, candidate to innovative target directed agents or to combined schedules including radiotherapy (RT) and chemotherapy (CT), since the accrual of this type of patients in clinical trials is limited.

## Methods/design

The ONCO-Aging study is part of a larger project (AGING Project) funded by the Italian Ministry of University and Research (MIUR), which has awarded a number of university departments as “departments of excellence” for research. The AGING Project is a project of excellence launched by the Department of Translational Medicine at the Università del Piemonte Orientale (UPO), Novara, IT, aimed to provide effective solutions to the emerging scientific and social challenges brought by aging. This project is based on four main pillars: (i) interdisciplinary approach, (ii) bench-to-bedside research, (iii) support to research and education, and (iv) public engagement. The AGING Project includes the activation of a biobank. The biobank is a research facility established by UPO to foster an inclusive scientific community where citizens, researchers and institutions come together to actively participate in the research process. The AGING Project will use the UPO biobank for the “Novara Cohort Study,” a study aimed to investigate the aging processes in the population residing in the Novara area and to identify lifestyles leading to healthy aging and risk factors associated with aging-related diseases.

### Study Design

**Fig. 1:**
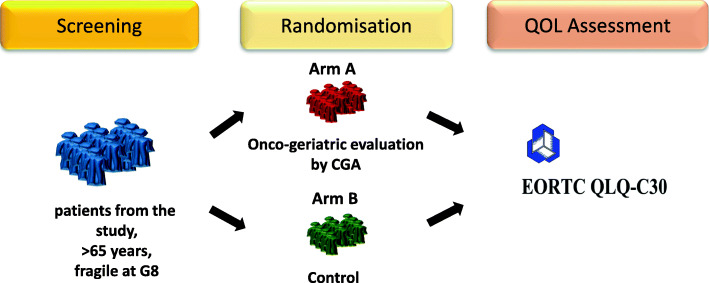
Prospective, observational, randomized study. Patients will enter the trial after completion the screening phase. Only patients aged ≥ 65 years and whose resulted as fragile using G8 screening tool with a score ≤ 14/17, will be randomized into 2 arms; Arm A: will be evaluated by Comprehensive Geriatric Assessment (CGA); Arm B: Control group. Both arms will be evaluated for their quality of life using EORTC – QLQ-C30 questionnaire, aiming to develop an accurate index for elderly patients with onco-hematological tumors using the CGA as primary study endpoint

**Fig. 2:**
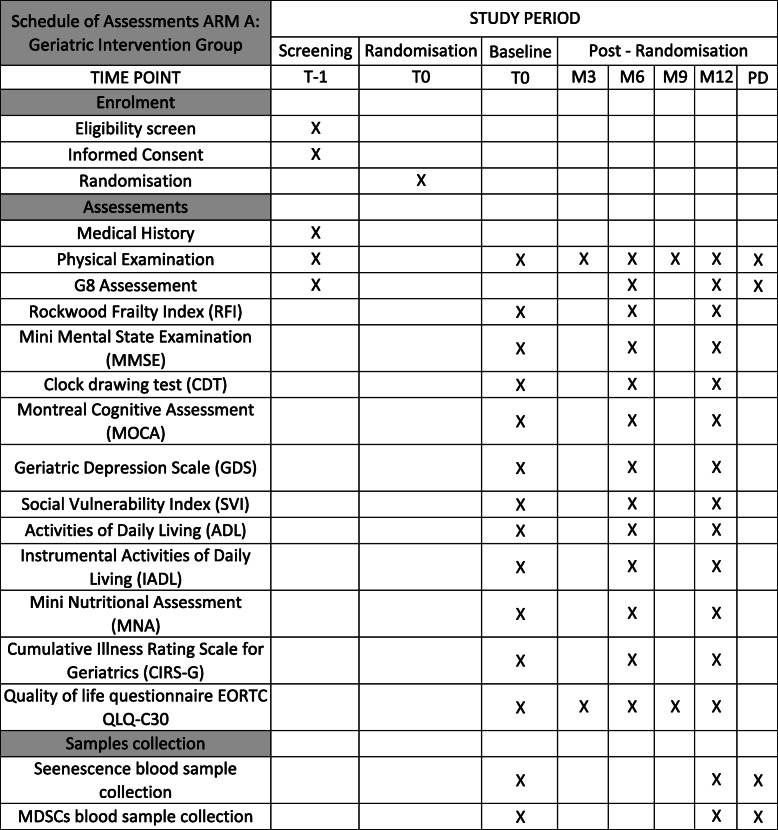
SPIRIT (Standard Protocol Items: Recommendations for Interventional Trials) diagram for ARM A: Geriatric Intervention Group; schedule of enrollment, interventions, and assessments. Abbreviations: M3; 3 months after randomisation, M6; 6 months after randomisation, M9; 9 months after randomisation, M12; 12 months after randomisation, PD; at progression

**Fig. 3:**
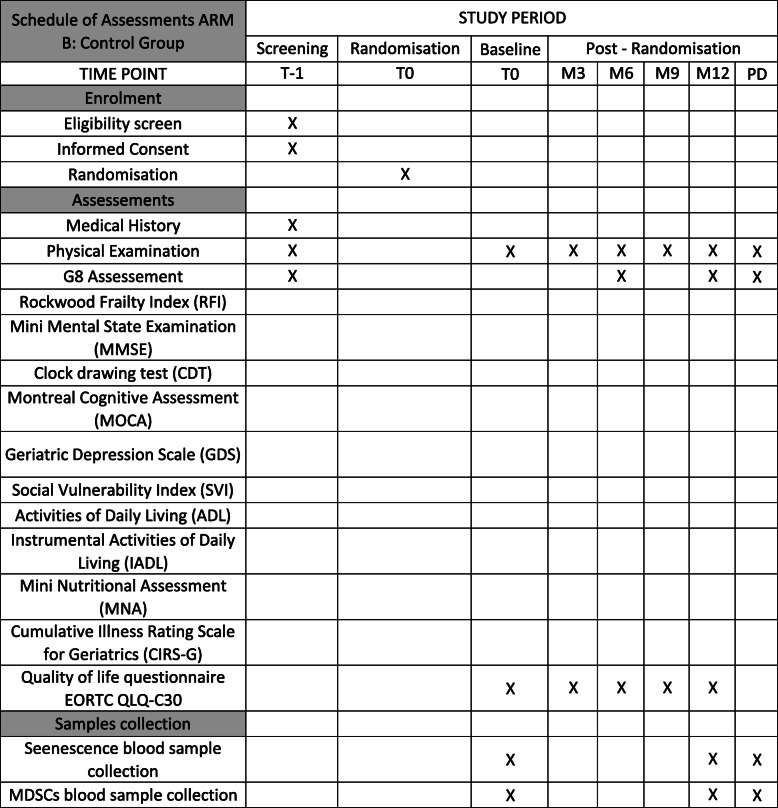
SPIRIT (Standard Protocol Items: Recommendations for Interventional Trials) diagram for ARM B: Control Group; schedule of enrollment, interventions, and assessments. Abbreviations: M3; 3 months after randomisation, M6; 6 months after randomisation, M9; 9 months after randomisation, M12; 12 months after randomisation, PD; at progression

The ONCO-Aging study is a prospective, randomized study and includes a clinical trial and a collateral biologic study. Patients older than 65 years, with hematological or oncological neoplasms, who refer for treatment to the Oncology, Hematology and Radiation Oncology Divisions of the University Hospital Maggiore della Carità in Novara, IT, and candidate to therapies with innovative target directed agents or to combined schedules including RT and CT, are screened for frailty by the G8 questionnaire; according to previous evidence, a score ≤ 14/17 is considered the threshold for frailty [16, 17]. Targeted directed agents include the following drugs: tyrosine-kinase inhibitors (TKis), monoclonal antibodies and immune checkpoint inhibitors (ICIs), alone or in combination schedules with CT. Combined treatment schedules including RT and CT are also included. Patients with a G8 score ≤ 14/17, are randomized in a 1:1 ratio to the following procedures: arm A, onco-geriatric evaluation by CGA; arm B, standard clinical care, according to local practice. Figure 1 shows the flowchart of the study. Patients are randomized by a REDCap dedicated system. Patients assigned to arm A undergo a CGA at baseline, before treatment start and every 6 months thereafter and may receive a CGA based geriatric intervention if required according to the geriatrician judgement (Fig. 2). Patients in arm B are managed according to clinical practice (Fig. 3). Blood samples to assess the prognostic and predictive value of cell-derived data (MDSC and T cell senescence) and their modification with treatment, will be collected at baseline and at the time of disease progression. Blood samples from healthy, age-matched controls, are recruited on a voluntary basis and also from the UPO Biobank.

### Objectives

The primary objective of the ONCO-Aging study is to assess the impact of the CGA and related interventions on the Quality of Life (based on the EORTC QLQ C30 questionnaire) of onco-hematological patients, with a G8 score ≤ 14/17, candidate to innovative target agents or integrated RT and CT schedules. Secondary objectives: *1.* to assess the percentage of treatment modifications, due to toxic events, in those patients who receive CGA assessment and related interventions, compared with those treated according to local clinical practice; 2) to evaluate the impact of CGA and related interventions on patient outcome and treatment adherence, as compared to those in the control arm.

The aim of the biological study is to assess the presence and the modifications induced by treatments of senescent T cell and MDSC in the peripheral blood of enrolled patients in both arms to evaluate the correlation with treatment efficacy and patient characteristics, including comorbidities, concomitant drugs, and lifestyle habits.

The primary endpoint is Health Related Quality of Life, based on the EORTC QLQ C30 questionnaire [18]; EORTC QLQ C30 questionnaire will be administered at baseline and every 3 months during treatment/follow up.

### Statistical design

The sample size was estimated by a two-tailed t-test (alpha = 0. 05, power = 0. 8 and an effect size Cohen (ES) equal to 0. 5) for a minimum clinically relevant difference of 10 points (SD = 20) on the global EORTC QLQ C30 Quality of Life scale. The planned sample size is 144 patients (72 per arm), considering a 10 % loss at follow-up. The primary endpoint will be evaluated on the ITT population. Baseline data will be summarized in terms of mean, standard deviation, median, I and III quartile, for continuous variables, and percentages for categorical variables. A test for the difference between two queued averages will be applied when comparing the total score for QoL. A level of significance of 5 % will be considered statistically significant. The study is expected to enroll about 144 patients with solid tumor neoplasms or hematological malignancies candidate to a first line treatment for advanced disease.

### Study Population

The ONCO-aging study was approved by the referral Ethical Committee (EC) of Maggiore della Carità Hospital, in Novara, IT, on December 18, 2019 (CE 230/19). Accrual in the ONCO-AGING trial started on January 14, 2020; study is actively recruiting. Main inclusion criteria are: age ≥ 65 years; diagnosis of advanced solid tumor or hematologic cancer at any stage, candidate to first line therapy with innovative target agents or combined RT and CT schedules; G8 score ≤ 14/17; signed IC. Patients with a life expectancy < 3 months are excluded.

### Study procedures

Comprehensive Geriatric Assessment (CGA): in arm A, patients receive a dedicated geriatric examination (60–90 min), administered by a dedicated geriatrician, and are classified according to CGA results and the Rockwood Frailty Index (RFI) as “frail” (RFI > 0.25), “unfit/prefrail” (0.08 > RFI < 0.25) or “fit” (< 0.08) [19].

The CGA includes evaluation of cognitive status with the Mini Mental State Examination (MMSE), Clock drawing test (CDT), and Montreal Cognitive Assessment (MOCA) [20]; of psychosocial status by the means of Geriatric Depression Scale (GDS) and Social Vulnerability Index (SVI) [21]; of disability by Activities of Daily Living (ADL) and Instrumental Activities of Daily Living (IADL) [22, 23]; of nutritional status by Mini Nutritional Assessment (MNA) [24]; of comorbidities by Cumulative Illness Rating Scale for Geriatrics (CIRS-G) and of pharmacological impact by number of drugs [25]; of sarcopenia and physical performance by Hand Grip Strength, Timed Up and Go test, Tinetti Scale, and number of falls in last six months [26]. CGA is performed at baseline and every 6 months. If issues are found in the areas of fragility, mid-term evaluations are foreseen, as indicated by the geriatrician. CGA based interventions will cover nutritional imbalance, social issues including the availability of caregiver and psychological interventions by dedicated psyco-oncologists.

**Fig. 4:**
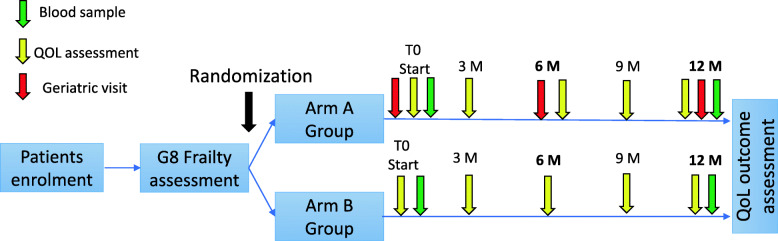
Study assessments timepoints. Patients will be randomized into 2 arms after completion of the G8 screening phase; Arm A; will be evaluated by Comprehensive Geriatric Assessment (CGA) at baseline and would be re-evaluated after every 6 months and If necessary, an onco-geriatric follow-up will be carried out. Arm B is the Control group, no geriatric visit is scheduled. Both arms will be evaluated for their quality of life using EORTC – QLQ-C30 questionnaire every 3 months. Blood samples from patients will be collected to evaluate T cells senescence and myeloid-derived suppressor cells at baseline and after 12 months or at disease progression

In both arms, QoL assessment by the standardized EORTC QLQ-C30 questionnaire is performed at baseline (before treatment start) and every 3 months. Disease evaluation is performed in both arms according to local clinical practice. Patients are observed for up to 12 months or until disease progression (Fig. 4).

### Biological study

The study is designed to evaluate T senescent cells and MDSC, and their modifications with treatment, in the peripheral blood of patients enrolled in the ONCO-aging study; blood samples will be collected at baseline (before start of therapy) and at the time of disease progression.

Blood samples (10 ml whole blood in Vacutainer EDTA tubes) are processed and analysed at the Immuno-Oncology Laboratory, at the Center for Translational Research on Autoimmune & Allergic Diseases – UPO-CAAD, in Novara, IT.

Peripheral mononuclear blood cells (PBMCs) are collected and isolated by Ficoll/Hypaque density gradient. T CD3 + Lymphocytes are isolated from the PBMCs by magnetic beads. The following markers will be tested by reverse transcription quantitative PCR, according to a manufactory protocol: p16INK4a and p21Cip/Waf1.

The Enzyme-Linked ImmunoSorbent Assay (ELISA) is used to evaluate the expression of cytokines and chemokines (e.g. CCL2/MCP-1, IFNγ, IL-6, IL-8), in plasma obtained from the same blood samples.

MDSC analysis is performed by flow cytometry in PBMCs. Assessment of MDSC phenotype is characterised by using the following markers: for monocytes-MDSC CD14^+^HLA-DR^+^, and for granulocytes-MDSC CD15^+^CD33^int^ and LOX-1^+^. Biological markers for T cell senescence and MDSC characterization are detailed in Table 1.

**Table 1:** Senescent T cells and Myeloid Derived Suppressor Cell subtypes markers

Type of cell	Markers
Senescent T cell	CD28- (co-stimulatory) CD57+ (senescence) KLRG1+ (senescence) p16INK4 (senescence) p21Cip/Waf1 (senescence)
M-MDSC	CD14+ (monocyte differentiation) CD11b+ (migration) HLA-DR^low/−^ (proliferation) CD15- (migration)
PMN-MDSC	CD14- (monocyte differentiation) CD11b+ (migration) HLA-DR+ (proliferation) CD15+ (migration) CD33^int^ (adhesion) LOX-1+ (proliferation)

Legend-*CD* Cluster of Differentiation; *PMN-MDSC* PolyMorphoNucler-Myeloid Derived Suppressor Cell; *M* Monocytic-Myeloid Derived Suppressor Cell; *LOX-1* Lectin-like OXidized low-density lipoprotein (LDL) receptor-1

## Discussion

Elderly patients present specific age-related conditions that may increase the risk of complications from anti-tumor therapy and compromise prognosis and QoL [7]. The ONCO-Aging trial is designed to assess whether the inclusion of a CGA and CGA based interventions, results into an amelioration of QoL, in onco-hematological patients receiving innovative target agents or treatment schedule including RT and CT. The study is dedicated to elderly patients with a G8 score ≤ 14/17. This aspect is particularly important in order to balance efficacy and tolerability of modern anticancer therapies, in particular TKis, ICIs and monoclonal antibodies, in old and frail patients, often excluded from registrative clinical trials, due to stringent inclusion criteria or physician fear of unexpected toxicities.

At present, most of the oncological and hematological treatments are established on the basis of the performance status (PS) evaluation, according to the World Health Organization (WHO) scale or to Eastern Cooperative Oncology Group (ECOG); this aspect is particularly evident in the inclusion criteria of clinical trials. However, this approach may not be enough to correctly evaluate an elderly patient, as severity of co-morbidities and frailty, may not directly correlate with PS alteration [27]. Thus, more specific functional scales, as those employed in the geriatric assessments, are needed to better define the clinical condition of elderly patients in the context of a CGA, with the aim to identify and separate elderly fit patients from those who are unfit. At present, there is no consensus on the exact definition of “unfit patient”, due to limited evidence, non-homogeneous inclusion criteria and inconsistent results.

From a biologic point of view, aging correlates with a progressive deterioration of the immune system in a process that is defined “immune senescence” [28], due to the associated functional decline of the immune system. Immune senescence is the result of many factors, including tissue damage, DNA damage, chronic inflammation, cell stress, cancer itself and anti-cancer treatments. In this context, an increasing number of T-cells and MDSC with a potential cytotoxic and suppressive activity, has been reported in observations from patients and preclinical data [29].

Likewise, it can be speculated that the biological age measured by the CGA and expressed in terms of fitness, unfitness and frailty could be related to a progressive inefficiency of the immune system, that might be predominant in frail patients. This hypothesis derives from the observation that in patients with solid and hematological malignancies, CGA-defined frail patients seem not to benefit from potentially life-saving therapies compared with younger counterparts [20]. Many studies provided evidence that the unfavorable outcome of elderly patients is partly related to clinical and biological differences in the disease itself, such as a more aggressive phenotype, more frequent advanced stage and presence of symptomatic disease [30]. To clarify this aspect, we added a biological aspect in our study to evaluate the role of T cell senescence and MDSC in our cohort of frail patients and to investigate the possible relationship among age, immune senescence and frailty.

Our trial has some limitations. It is a monocentric exploratory study, with a limited number of patients with different solid and hematologic tumors. The different types of diseases that can be included usually have different timepoints for the radiological and clinical evaluations, but as a starting protocol, we decided to identify standard timepoints visits for geriatric and radiologic evaluations, according to local clinical practice and guidelines. On the other side, given the network organization of the “Rete Oncologica Piemontese”, where the University Hospital Maggiore della Carità in Novara, IT, is the second most important hub in the region, and therefore is allowed to prescribe innovative oncological treatments and to perform aggressive combination therapies with the association of RT and CT, the probability of poor compliance or drop out during follow up is minimal. This eventuality is also further reduced by the regular introduction of palliative simultaneous care in frail patients of any age, being our center an ESMO designated center for simultaneous care. The results of the ONCO-aging study, dedicated to the elderly patient population, will be used to define a global strategy within the “UPO AGING project”, to improve our aging-specific research with a multicenter study including a larger number of patients and a more selective division by cancer type and treatments.

### Trial status

The first patient was screened by G8 in December 2019, after EC approval and randomized on January 2020. The planned recruitment period is 2 years. Trial enrolment was halted during the COVID-19 emergency period in Italy (from March to July 2020). Currently, the study is actively recruiting; 358 patients have been screened by G8 so far, and 63 have been randomized.

## Data Availability

The datasets generated and analysed during the current study will be made available once the study is completed upon reasonable request of the corresponding author.

## Abbreviations

QoL: Quality of life
CGA: Comprehensive Geriatric Assessment
eCRF: Electronic Case Report Form
PI: Principal Investigator
OS: Overall Survival
G8: Geriatric 8
BMI: Body Mass Index
PCR: Polymerase Chain Reaction
PB: Peripheral Blood
EDTA: Ethylenediaminetetraacetic acid
NK: Natural Killer
FC: Flow cytometry
PBMC: Peripheral Blood Mononuclear Cell
FACS: Fluorescent-Activated Cell Sorter
PD: Progressive Disease
DPO: Data Protection Officer
SASP: Senescence-Associated Secretory Phenotype
ITT: Intention-To Treat
